# Blinded by Zika? A missed HIV diagnosis that resulted in optic neuropathy and blindness: a case report

**DOI:** 10.1186/s13104-017-2970-5

**Published:** 2017-12-01

**Authors:** Tiffany Hirschel, Heimo Steffen, Victor Pecoul, Alexandra Calmy

**Affiliations:** 1 Division of Infectious Diseases, Geneva’s University Hospitals, 4 Rue Gabrielle Perret-Gentil, 1211 Geneva 14, Switzerland; 2 Division of Emergencies, Geneva’s University Hospitals, 4 Rue Gabrielle Perret-Gentil, 1211 Geneva 14, Switzerland; 3 Division of Ophthalmology, Geneva’s University Hospitals, 4 Rue Gabrielle Perret-Gentil, 1211 Geneva 14, Switzerland; 4 Department of General Medicine, Geneva’s University Hospitals and Faculty of Medicine, Geneva, Switzerland; 5 Department of Clinical Neurosciences, Geneva’s University Hospitals and Faculty of Medicine, Geneva, Switzerland; 6 Department of Medicine Specialities, Geneva’s University Hospitals and Faculty of Medicine, Geneva, Switzerland

**Keywords:** Optic neuropathy, Primary HIV infection, Atypical HIV infection

## Abstract

**Background:**

Typical symptoms of an acute human immunodeficiency virus (HIV) infections like fever and rash are not specific and can be caused by a multitude of other pathogens, such as Zika or rickettsiosis. Up to 30% of primary HIV infection do not present with the typical flu-like symptoms and thus represent a diagnostic challenge. In this report, we describe a rare case of optic neuropathy as the initial presentation of primary HIV infection, which resulted in irreversible blindness. To our knowledge, only four cases of optic neuropathy resulting from a recent HIV seroconversion have been reported.

**Case presentation:**

In January 2015, a 72-year-old man presented with a rash, fever and diffuse myalgias after returning from a fortnight in Cuba. In the context of the current polemic, Zika was considered likely. A diagnostic work-up, including dengue fever and Zika, was negative. Symptoms resolved spontaneously. In March, the patient experienced a sudden loss of vision first on one, a few days later on the other eye. Magnetic resonance imaging showed optic nerve enhancement suggesting neuritis. Numerous infective causes were sought and the patient was diagnosed with HIV. Corticosteroids and antiretroviral therapy were initiated but vision did not improve. Four weeks later an optic atrophy developed. After more than a year of follow-up the patient remains blind. Stored serum from January revealed a detectable viremia with a negative Western blot assay, typical of acute HIV infection.

**Conclusions:**

Optic neuritis is a rare complication of early HIV infection. Only four others cases have been described, some of which recovered their vision after the administration of corticosteroids and/or ARV treatment. The balance between ischemic and neuroimmune processes may play a role in recovery. Delayed diagnosis, due to an unjustified focus on the Zika virus may have contributed to the tragic outcome.

## Background

Typical symptoms of primary human immunodeficiency virus (HIV) infection are fever, malaise/fatigue, pharyngitis and/or a rash. These symptoms are not specific and can occur in a multitude of other infections, such as Zika [1]. However, Braun et al. [2] report that approximately 30% of these patients do not present with typical symptoms and may represent a diagnostic challenge. Atypical presentations include no symptoms at all, acute psychiatric disorders, acalculous cholecystitis and severe encephalopathy [2]. Overall, only one-fifth of symptomatic primary HIV infections are caused by opportunistic infections, all others are due to the effect of the virus itself [2].

Valcour et al. showed that HIV neurotropism can occur as early as 8 days after transmission causing central nervous system (CNS) inflammation [3]. According to a cohort study by Helmuth et al. [4], approximately 1 in 2 patients with symptomatic primary HIV infection presents with neurological symptoms. Fulminant neurologic involvements, such as meningitis or encephalitis, have been described in association with acute HIV infection [5–7]. Peripheral nerve involvement, such as facial palsy, oculomotor palsy or vestibular neuritis have also been observed [8–10]. To our knowledge, only four cases of optic neuropathy resulting from a recent HIV seroconversion have been reported [11–14]. We describe a rare case of optic neuropathy as the initial presentation of primary HIV infection, which resulted in irreversible blindness.

## Case presentation

**Fig. 1:**
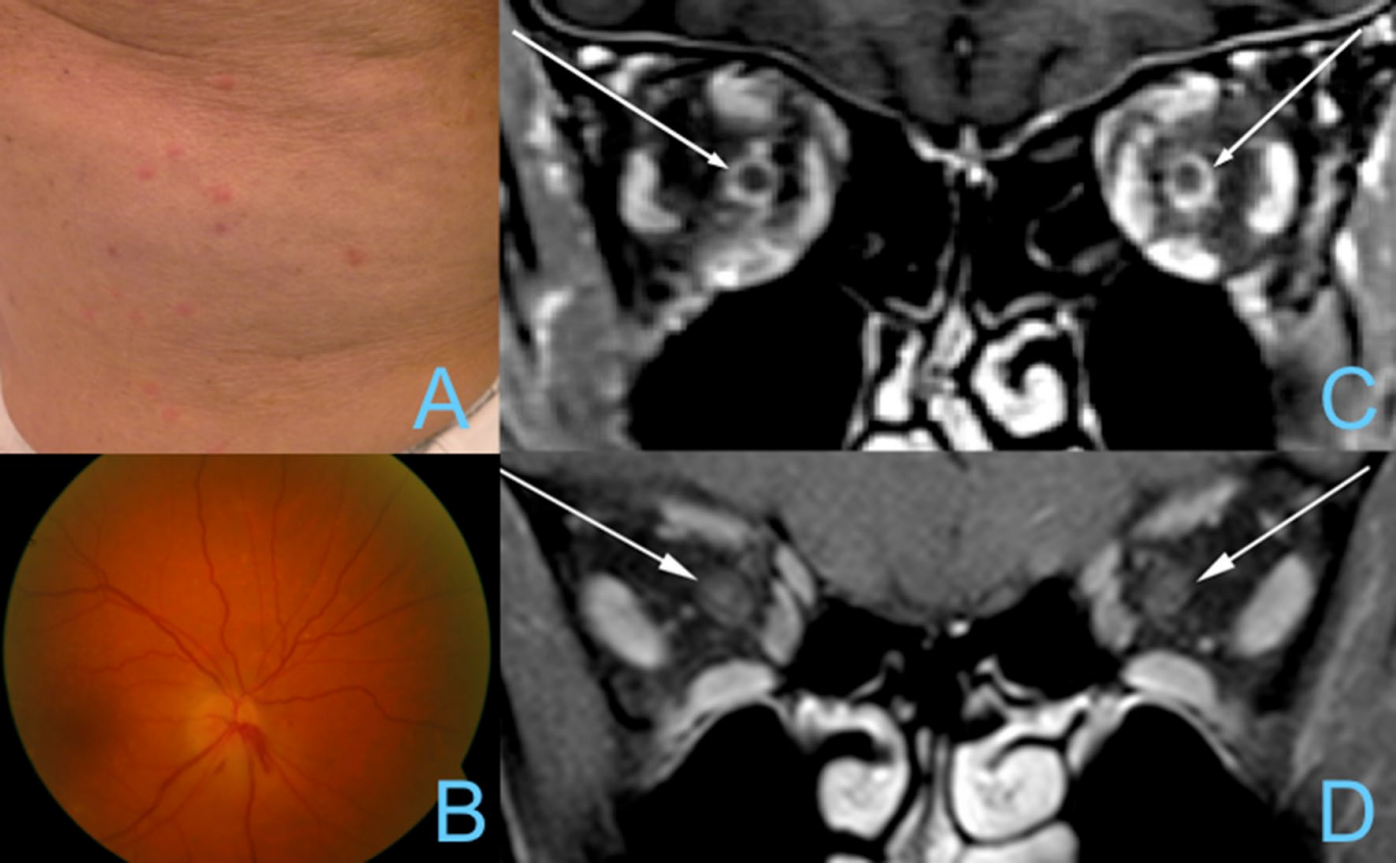
**A** Papular rash; **B** right optic nerve oedema; **C** patient’s optic nerve enhancement on MRI; **D** normal optic nerve as comparative

In January 2016, a 72-year-old man with no notable medical history presented at our emergency center for tropical diseases a few days after returning from a 2-week vacation in Cuba. He described skin lesions without pruritus, fever and diffuse myalgia. Clinical examination revealed a diffuse papular rash on his arms, legs and trunk (Fig. 1A) and two ulcerated lesions on the oropharynx. Considering his recent travel and no sexual risk history, the most likely diagnosis was a tropical infection. Considering the current polemic and excitement, Zika infection was considered as a likely hypothesis. The patient was screened for Zika, dengue and rickettsiosis and put on a 10-day course of empiric antibiotic treatment with doxycycline. The skin rash disappeared completely within 2 weeks, but the laboratory results did not confirm the hypothetic diagnosis.

In March 2016, he presented to the ophthalmic emergency department with a rapidly evolving, painless, bilateral loss of vision. Within a few days, his visual acuity dropped down to 0.1 in the right eye and 0.05 in the left eye. without any other accompanying symptoms (no ocular pain, neurological symptoms, headache or scalp tenderness, jaw claudication, fever, proximal myalgia, arthralgia or fatigue). He reported not having been exposed to any toxins, drugs or vaccines in the weeks preceding the loss of vision. Fundoscopic examination revealed bilateral papillary edema with thunder hemorrhages in the right eye (Fig. 1B). Goldmann’s visual fields showed an altitudinal superior deficit in the right eye and a large central scotoma in the left eye with a small preserved inferior temporal area. He was hospitalized and immediately started on high doses of corticosteroids and aspirin. Fluorescein angiography of the retina excluded arterial leakage or obstruction. Orbital magnetic resonance imaging (MRI) revealed an increase of the signal of the right optic nerve and the appearance of a hypersignal on the left on the STIR sequence, associated with an increase in the spiculous and irregular contrast intake of the intraconical fat around the sheaths of the optic nerves. This aspect confirmed the clinical suspicion of optic neuritis (Fig. 1C, D).

Considering the large differential diagnosis of optic neuritis, multiple tests were performed. Blood cultures showed no leukocytosis or raised C-reactive protein or erythrocyte sedimentation rates. Vitamins B1, B6 and folic acid were normal. B12 was found to be decreased at 133 pmol/L, which was not sufficient to explain the clinical picture. Infective causes of optic neuritis, such as human cytomegalovirus, varicella zoster virus, herpes simplex virus type-1, toxoplasmosis, Lyme disease, cat scratch disease, syphilis, Epstein–Barr virus and tuberculosis were all excluded either by serology or by cerebrospinal fluid polymerase chain reaction. Autoimmune disorders were also investigated and excluded by measuring autoimmune markers, such as antinuclear antibodies, antinucleoproteins, antiphospholipids, antineutrophil cytoplasmic antibodies, human leukocyte antigen-B51, cryoglobulin and antiaquaporin anti-bodies and anti MOG anti-bodies.

**Fig. 2:**
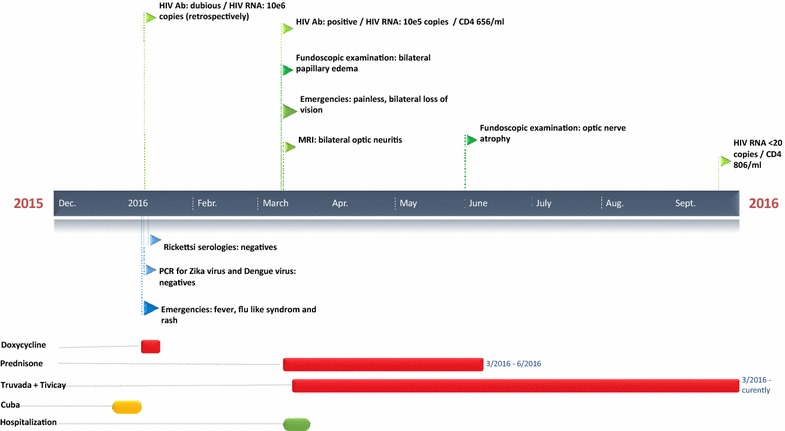
Timeline

Finally, an HIV infection with a viral load of 1.9E^5^ copies/mL in the serum and 4.8E^3^ in the cerebrospinal fluid was diagnosed. The CD4 count was 656 per mm^3^. A stored serum from 3 months previously was analyzed retrospectively and showed an HIV viral load of more than 10^6^ copies/mL with a negative western blot. Based on these findings, the diagnosis of an acute retroviral syndrome was retained and antiretroviral (ARV) treatment with dolutegravir (Tivicay^®^, GlaxoSmithKline), tenofovir and emtricitabine (Truvada^®^, Gilead Sciences) was started a few days after presentation. Although HIV-RNA was rapidly undetectable with a CD4 cell count above 700 cells/mm^3^, the patient’s visual acuity did not improve. Five months after ARV treatment initiation and corticosteroid therapy, his visual acuity was 0.05 on the right and 0.02 on the left eye, respectively. Fundoscopic examination showed a marked atrophy of the optic disc. Optic coherence tomography showed a marked loss of retinal nerve fiber thickness throughout the entire optic disc, confirming the marked loss of retinal nerve fiber layer ganglions. The case is summarized in Fig. 2.

## Discussion

The term “optic neuropathy” refers to optic nerve damage, which can be caused by a multitude of disorders including demyelinating, infectious, inflammatory and ischemic diseases and mechanical compressive lesions [15]. A toxic etiology or malnutrition are less common. In patients with HIV infection, optic neuropathy is most frequently due to opportunistic pathogens and/or secondary to retinal inflammation when patients are immunocompromised, e.g. after a longstanding HIV infection [16]. In patients with preserved CD4 cells, ocular manifestations resulting from acute HIV infection are extremely rare.

To our knowledge, only four cases have been reported of acute optic neuropathy related to a new HIV infection (Table 1) [11–14]. The delay between viral transmission and visual loss ranged from 10 days [12] to several months. Clinically, two different types of optic nerve involvement could be distinguished: inflammatory and ischemic. The prognosis of inflammatory optic neuropathy, i.e. optic neuritis, is usually good.

**Table 1:** Literature review summary of the patient case’s main characteristics

	Age and sex	Symptoms preceeding loss of vision (LOV)	Time between suspected time of infection and LOV	LOV	MRI enhancement	HIV viral load (VL) copies/mL and CD4/mm^3^	Treatment	Evolution	Relapse
This case	72 years ♂	Flu like symptoms	3 months	Bilateral	Yes	VL: 1.9E5 CD4: 656	Corticosteroids ARV	No improvement	No
Coriat et al. [11]	57 years ♂	Flu like symptoms Bilateral peripheral facial paralysis T6–T9 Dysesthesia	2 months	Bilateral	No	VL: 1.5^E^5 CD4: 388	Corticosteroids ARV	No improvement	No
Larsen et al. [12]	21 years ♀	Flu like symptoms Headache Photophobia	10 days	Bilateral	Yes	VL: 1.3^E^5 CD4 558	Corticosteroids	Complete resolution	No
Graber et al. [13]	28 years ♀	Paresthesia left arm	10 weeks	Bilateral	Unknown	VL: 669 CD4: 554	Corticosteroids	Complete resolution	Yes
Nkoghe et al. [14]	32 years ♂	None	6 months	Unilateral	Unknown	VL: 1.4^E^3 CD4 687	Corticosteroids	Complete resolution	Yes

Larsen et al. [12] and Graber et al. [13] described two cases of rapidly progressive bilateral loss of vision after recent HIV infection. Larsen reported that the MRI demonstrated thickening of both optic nerves and marked signal enhancement after intravenous injection of gadolinium-dtpa in the context of a demyelinating neurological disorder. In Graber’s case, vision loss was preceded by a paraesthesia of the left arm. A computed tomography (CT) brain scan showed a ring-enhancing solitary hypodense lesion in the right temporo-parietal region. Biopsy of this region revealed a demyelination, similar to multiple sclerosis, with preserved axons, inflammatory infiltration with multiple macrophages, and reactive astrocytes. In these 2 cases, corticosteroids were beneficial at first presentation, but less so during a relapse in 1 case [13] and plasmapheresis was successfully used.

Non arteritic ischemic optic neuropathies (NAAION) are caused by a lack of balance between perfusion pressure and vascular resistance and prognosis is poor overall. To our knowledge, only one case of NAAION has been hypothetically linked to primary HIV infection [11]. Coriat reported painless loss of vision with altitudinal visual field deficit, no optic nerve enhancement on MRI, and no recovery after corticosteroids or ARV treatment, all suggestive of an ischemic origin. Increased risk of cardiovascular events and cerebral vasculopathy has been linked to chronic HIV infection where a link between acute HIV infection and ischemia has not been described. The physiopathological mechanism is unclear [11, 12].

Nkoghe et al. [14] described a case of unilateral vision loss that responded well to corticosteroids. Initially, the brain CT scan was normal and MRI was not performed. Three months later, the patient developed frontal symptoms and memory loss. HIV antibodies became positive and MRI showed three nodules in white matter. Histological examination demonstrated inflammation stigma with necrosis, demyelination, but also the destruction of axons that could be compatible with the demyelinating process of the central nervous system. After the introduction of ARV therapy, neurologic symptoms and MRI lesions regressed and there was no relapse during a follow-up of 4 years.

Optic neuritis is characterized by a rapidly progressive loss of vision with optic nerve contrast enhancement on MRI. It is a form of neuroimmune disorder and can be triggered by many types of infection, particularly viral infections. Several other neuroimune disorders, such as multiple sclerosis [17], transverse myelitis [18] or acute disseminated encephalomyelitis [6] have been reported in the context of acute HIV infection in immunocompetent patients. The beneficial effect of ARV therapy in neuroimmune disorders has been observed at least once during acute HIV infection [5] and twice during chronic HIV [19, 20]. From these very few case reports, we suggest to start ARV therapy immediately when a neuroimmune disorder is possibly related to an HIV incident infection. Our case presented initially as an inflammatory optic neuropathy. However, the dramatic outcome, lack of clinical response to treatment and the atrophy that ensued may suggest an additional ischemic process. Our hypothesis is that the inflammatory or autoimmune response caused by HIV infection could lead to an ischemic damage of retinal ganglion cells by interfering with the vascular supply of the optic nerves. The papilledema (and the peripapillary hemorrhage) can be considered as a uniform clinical sign of a final common path resulting from damage of the axoplasmic flow by autoimmune response related ischemia generated a papillary edema important enough to compromise the optic nerve’s vascular supply and resulted in ischemia.

## Conclusions

In summary, atypical primary HIV infection represents a diagnostic challenge which clinical outcome may be devastating. Up to 83% of acute HIV infections are missed during first encounter [21]. As illustrated by this case report, primary HIV infection can present as an atypical neuroimmune disorder, such as isolated bilateral optic neuropathy, and result in severe and devastating consequences. It is impossible to say whether more timely treatment would have prevented the tragic outcome. This case is a reminder that in early HIV infection, autoimmune phenomena may produce impressive manifestations. Unfortunately, these will always remain difficult to explore because of their rarity and the lack of an appropriate animal model.

## Abbreviations

ARV: antiretroviral treatment
CNS: central nervous system
CT: computed tomography
DTPA: diethylenetriamine penta-acetic acid
HIV: human immunodeficiency virus
LOV: loss of vision
MOG: myelin oligodendrocyte glycoprotein
MRI: magnetic resonance Imaging
NAAION: non arteritic ischemic optic neuropathies
RNA: ribonucleic acid
STIR: Short TI Inversion Recovery
